# Association of handgrip strength asymmetry and weakness with functional disability among middle-aged and older adults in China

**DOI:** 10.7189/jogh.14.04047

**Published:** 2024-03-29

**Authors:** Quhong Song, Xiaoyu Shu, Yuxiao Li, Yanli Zhao, Jirong Yue

**Affiliations:** Department of Geriatrics and National Clinical Research Center for Geriatrics, West China Hospital, Sichuan University, Chengdu, Sichuan, China

## Abstract

**Background:**

Little is known about the association of handgrip strength (HGS) asymmetry with functional disability in China. We aimed to examine the individual and combined association of HGS asymmetry and weakness with functional disability among middle-aged and older Chinese adults.

**Methods:**

We included participants aged ≥45 years from two waves of the China Health and Retirement Longitudinal Study (2011 and 2015). HGS weakness was defined as the maximal HGS<28 kg for men and <18 kg for women. HGS asymmetry was measured by dividing the maximal nondominant HGS (kg) by the maximal dominant HGS (kg), with the value <0.90 or >1.10 considered as asymmetry. Functional disability was assessed by activities of daily living (ADL) and instrumental activities of daily living (IADL) and was defined as encountering difficulty in completing one or more ADL/IADL tasks. The logistic regression models were used to explore the association between HGS measures and functional disability.

**Results:**

11 950 (mean age 59.2 ± 9.6 years, 47.9% males) and 7540 (mean age 57.5 ± 8.6 years, 50.1% males) participants were included in the cross-sectional and prospective study, respectively. HGS asymmetry and weakness, individually or simultaneously, were associated with an increased prevalence of functional disability. During the four-year follow-up, 1822 (24.2%) participants had incident functional disability. The separate exposure to HGS asymmetry (odds ratio (OR) = 1.18; 95% confidence interval (CI) = 1.05–1.32) or weakness (OR = 1.59; 95% CI = 1.30–1.95) was independently associated with functional disability. For combined associations, those with both weakness and asymmetry showed the greatest risk of new-onset functional disability (OR = 1.91; 95% CI = 1.45–2.52).

**Conclusions:**

HGS asymmetry and weakness were associated with a higher risk of functional disability. Assessing HGS asymmetry together with weakness may help to better identify those at risk of functional disability to enable early interventions.

Functional disability, characterised by the impaired ability to perform activities required for independent living, affects around 1.3 billion people worldwide (16% of the global population) [1]. It is common among older adults, with an estimated 34.4% of the world’s population aged 60 years and above experiencing disabilities [1]. This number will continuously increase as the population ages [1]. Preserving the functional capacity that enables well-being in later life is the key to healthy aging and has become a top priority for promoting public health [2]. Functional disability can cause undesirable outcomes like depression [3], falls [4], cognitive impairment [5], hospitalisation [6], mortality [7], exerting heavy burdens on individuals, families, and society. Fortunately, functional disability is preventable and intervenable [8], and a better understanding of its underlying risk factors is vital to designing effective interventions to delay functional loss and associated negative consequences.

Adequate muscle strength is fundamental to maintaining physical functioning, and deficits in muscle function strongly predict disability [9,10]. Handgrip strength (HGS) is the simplest method for assessing muscle function in clinical practice, which can be easily measured with a handgrip dynamometer [11]. It has been reported that HGS weakness, as assessed by maximal HGS, was associated with functional limitations [10] and increased risk of other poor health outcomes (e.g. chronic morbidities, frailty, premature mortality) [12]. However, the maximal HGS alone cannot fully assess muscle function, as it exclusively involves grip force in one hand while disregarding hand dominance and hand differences [12]. The human body exhibits laterality, with a natural difference in strength between hands [13,14]. HGS asymmetry, featured by a large difference between bilateral grip strength, has recently been identified as another dimension of muscle impairment [15] and has important prognostic values [16,17]. Emerging evidence suggests that HGS asymmetry and weakness may have synergistic effects on health [18-21]. Analysing those two indicators simultaneously may provide a better understanding of how different aspects of muscle function influence health.

With respect to functional health, although some literature has reported the association of HGS asymmetry and weakness with functional disability [15,21–23], only a few have assessed their joint relationship with functional status [15,21]. In addition, previous studies focused primarily on Western countries, with limited evidence from Asian countries. Given the ethnic and geographical variations in anthropometric measures [24], whether and to what degree the relationship of HGS asymmetry and weakness with functional disability can be applied to non-Western populations remains to be elucidated. China hosts the largest older population in the world and is aging faster than many other countries [2,25]. In 2020, there were 264 million Chinese aged 60 years and over (18.7% of the total population) [26], and this population is estimated to reach 479 million (35.1%) by 2050 [27]. In parallel, it is projected that the number of disabled and semi-disabled elderly will increase from 48 million in 2020 to 121 million in 2050 [28]. With China's population aging, functional disability not only threatens the quality of life for millions of people, but also brings significant social, economic, and health care burdens given the growing demand for long-term care and medical services [2,28]. It is therefore of particular practical importance to identify relevant modifiable risk factors of disability in Chinese settings.

In the present study, we conducted the cross-sectional and longitudinal analysis to explore the individual and combined association of HGS asymmetry and weakness with functional disability among middle-aged and older Chinese adults, by using the nationally representative data from the China Health and Retirement Longitudinal Study (CHARLS). The main purpose was to evaluate the role of incorporating HGS asymmetry assessment on disability risk, and the findings may provide insights into early identification, prevention, and intervention of functional disability. We hypothesised that individuals with both HGS asymmetry and weakness will have a higher risk of functional disability.

## METHODS

### Study population

This study was a secondary data analysis based on the CHARLS, which is a nationally representative prospective survey of Chinese community-dwelling adults aged 45 years and older. A detailed description of CHARLS has been previously published [29]. Briefly, a total of 17 708 participants in 10 257 households were recruited from 150 counties or districts and 450 villages within 28 provinces in China between June 2011 and March 2012 (wave 1). Subsequent follow-ups were conducted biennially, with the first follow-up survey in 2013 (wave 2), the second in 2015 (wave 3), and the third in 2018 (wave 4). The CHARLS data sets are publicly available at http://charls.pku.edu.cn/en. This study followed the Strengthening the Reporting of Observational Studies in Epidemiology (STROBE) reporting guideline [30].

**Figure 1:**
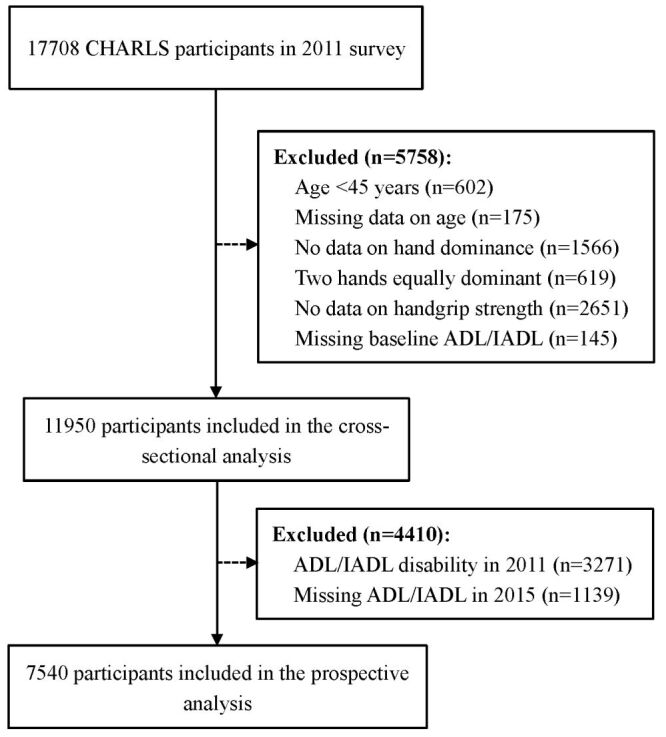
Flowchart of the participants’ selection. ADL – activities of daily living, IADL – instrumental activities of daily living

In the current study, we used two waves of CHARLS data (2011 and 2015). Eligible participants were those aged ≥45 years. We excluded subjects if they had: (1) missing values on age; (2) no information on hand dominance, or with two hands equally dominant; (3) no data on HGS; and (4) missing data on baseline functional status. The study was divided into two parts: (1) In the cross-sectional analysis, we eliminated 5758 participants following the above-predefined exclusion criteria, leaving 11 950 participants for the cross-sectional analysis. (2) In the longitudinal analysis, we further excluded 3271 subjects with baseline disability in 2011, and 1139 individuals with missing information on functional status in 2015, which resulted in 7540 eligible participants. The detailed flowchart of participants’ selection process was displayed in **Figure 1**.

### Handgrip strength weakness and asymmetry

HGS (kg) was measured by a mechanical dynamometer (Yuejian^TM^ WL-1000, Nantong, China), which has been proven to have satisfactory test-retest reliability [17]. After reporting hand dominance, participants performed the test in a standing position with their elbow flexed at 90° and held the dynamometer as hard as possible. For those who cannot stand unaided, HGS can be measured in a sitting position. Each participant was tested twice for each hand. If the subject was unable to perform the task on either hand (i.e. the greatest HGS value in one hand equals zero), the HGS was treated as missing. The maximum value of all four readings was used to evaluate HGS weakness. According to the Asian Work Group for Sarcopenia 2019 consensus [31], HGS weakness was defined as the maximal HGS of <28 kg in men and <18 kg in women.

HGS asymmetry was assessed by the HGS ratio, which was determined by dividing the maximal HGS of the nondominant hand (kg) by that of the dominant hand (kg) [20,21]. Consistent with prior literature [13], the 10% rule, which suggests that HGS is generally 10% stronger in the dominant hand compared with the nondominant hand, was employed to define HGS asymmetry. Accordingly, participants with an HGS ratio <0.90 or >1.10 were considered to have HGS asymmetry.

Based on HGS weakness and asymmetry categories, we further divided participants into four groups: (1) normal and symmetric HGS, (2) asymmetry only, (3) weakness only, and (4) weakness and asymmetry.

### Assessment of functional status

The primary outcome was functional disability, and the secondary outcome was functional dependency. Functional disability was measured by activities of daily living (ADL) and instrumental activities of daily living (IADL). ADL includes six activities: eating, dressing, bathing, toileting, getting in or out of bed, and controlling urination and defecation [32,33]. IADL consists of five activities: shopping, cooking, doing housework, taking medications, and managing money [32,33]. Each item in ADL and IADL contains four answers: ‘don’t have any difficulty’, ‘have difficulty but can still do it’, ‘have difficulty and need help’, and ‘cannot do it’, which were assigned scores from 0 to 3, respectively [33]. Those with difficulty in one or more ADL/IADL items were classified as having functional disability [32,33]. We also calculated the continuous scores of functional disability (scores 0-33), ADL disability (scores 0-18), and IADL disability (scores 0-15), with higher scores indicating poor function [33].

Functional dependency was determined by the previously reported interval-of-need method [34], which is considered to be a more informative measure of disability burden that can reflect the potential need for personal and family care, supervision, and medical-related services. We divided participants into four categories based on the frequency with which they need care: high dependency (requires 24-hour care), medium dependency (requires help at regular times daily), low dependency (requires help less than daily), or independent (don’t need care) [34,35]. In line with prior studies [35,36], we constructed the interval-of-need categories according to the need for help with ADL and IADL activities (Table S1 in the **Online Supplementary Document**).

### Covariates

The following baseline variables were included as potential covariates: age (years), sex, marital status (married/partnered, unmarried/others), educational level (illiterate, primary school, middle or high school, college or above), residence (urban, rural), household income per capita (Chinese yuan (CNY¥)), occupation (agricultural work, non-agricultural work, retired, unemployed or never work), social activities (no, yes), smoking status (never, past, or current), drinking status (never, ≤1 time/mo, >1 time/mo), multimorbidity (the presence of 0, 1, and ≥2 chronic diseases), visual problems (no, yes), hearing problems (no, yes), depression symptoms, cognitive function, and body mass index (BMI).

In this study, social activities were defined as the respondent’s engagement in any of the following activities: interacting with friends; playing mahjong, chess, cards, or going to a community club; going to dance, exercise, practice Qigong in parks or other places; taking part in a community-related organisation; doing voluntary or charity work; and attending an educational or training course. Multimorbidity was assessed based on the presence of 14 chronic conditions: hypertension, diabetes, heart problems, stroke, dyslipidemia, cancer, chronic lung disease, digestive disease, liver disease, kidney disease, arthritis, emotional and mental problems, memory-related disease, and asthma. Depression symptoms were measured by the 10-item Center for Epidemiologic Studies Depression Scale, yielding a total score of 30 [37]. Cognitive function was evaluated by episodic memory, the Telephone Interview of Cognitive Status (TICS-10), and figure drawing, totaling a score of 21 [38]. BMI was calculated as weight divided by the square of height (kg/m^2^) and was classified into four groups: underweight <18.5, normal 18.5–23.9, overweight 24.0–27.9, and obese ≥28.0 kg/m^2^.

### Statistical analysis

Continuous variables were described as mean with standard deviation (SD) or median with interquartile range (IQR), and categorical variables were presented as frequency with percentages. For comparison among different groups, χ^2^ or Fisher exact test was used for categorical variables; whereas the *t* test, Mann-Whitney U test, analysis of variance, or Kruskal-Wallis H test was used for continuous variables, as appropriate. Missing covariates for participants (55.7% (6662/11950)) and 54.2% (4088/7540) in the cross-sectional and prospective analysis, respectively) were assumed to be randomly missing and were imputed using the chained equations method with 10 replications via the ‘mi estimate’ command in Stata statistical software version 15.0.

The binary logistic regression models were used to assess the cross-sectional and longitudinal association of HGS status with functional disability, and the multinomial logistic regressions were employed to examine the associations of HGS status with functional dependency (the independent group as reference). The results were reported as odds ratio (OR) and 95% confidence interval (CI). Relations of the exposures with functional outcomes were first explored by the separate exposure of HGS asymmetry and weakness. Then, the models were re-run using the composite measure derived from the two exposures. Three models were fitted: (1) model 1: unadjusted; (2) model 2: age and sex-adjusted; (3) model 3: further adjusted for marital status, educational level, residence, household income, occupation, social activities, smoking, drinking, multimorbidity, visual problems, hearing problems, depression score, cognitive function, and BMI. In the prospective analysis, follow-up time was also adjusted in model 3.

Given that age and sex may affect the association between HGS status and functional disability [21,39], subgroup analyses were performed to explore the possible heterogeneous effects of age (<60/≥60 years) and sex. In addition, as HGS difference between hands may be influenced by hand dominance [14,40], which may potentially bias the association of HGS measures with functional outcomes [21], additional stratified analysis by hand dominance (right-handed/left-handed) was further conducted. The potential interactions were evaluated by including a multiplicative interaction term in the model. The results were visualised by forest plots. Three sensitivity analyses were conducted to test the robustness of our findings: (1) we repeated the analyses using the complete-case data sets (5288 and 3452 participants in the cross-sectional and longitudinal analyses, respectively) to evaluate the potential effect of multiple imputations; (2) as HGS between hands may vary across individuals [14], to reduce asymmetry misclassifications, we also defined HGS asymmetry using 20 and 30% cut-points and re-ran the models. Specifically, asymmetry was defined as HGS ratio <0.80 or >1.20 (20%), or HGS ratio <0.70 or >1.30 (30%) [19,22,23]; (3) we reanalysed the data treating the outcomes as continuous disability scores, using linear regression models, to assess the robustness of the relationship between HGS status and functional disability.

All analyses were performed with Stata 15.0 (StataCorp, College Station, TX, USA), and a two-sided *P* < 0.05 was considered statistically significant.

## RESULTS

### Baseline characteristics

**Table 1** presented the baseline characteristics of the study population in the cross-sectional analysis. The mean age of the 11 950 participants was 59.2 ± 9.6 years, and 5729 (47.9%) were males. Of these subjects, the prevalence of HGS weakness and asymmetry was 10.5% (1256/11 950) and 41.6% (4974/11 950), respectively. Compared with individuals without weakness or asymmetry, those with weakness or asymmetry were more likely to be older, unmarried, less educated, and had higher comorbidity burden, higher depression score, and lower cognitive score (all *P* < 0.001, **Table 1**). Baseline characteristics by the combined HGS weakness and asymmetry categories were provided in Table S2 in the **Online Supplementary Document**. Meanwhile, Table S3–S4 in the **Online Supplementary Document** also showed the characteristics of 7540 participants without baseline functional disability (mean age 57.5 ± 8.6 years, 50.1% men) in the longitudinal study.

**Table 1:** Baseline characteristics of 11 950 participants by HGS status in the cross-sectional analysis

Characteristics	Total (n = 11 950)	HGS weakness			HGS asymmetry		
		**No (n = 10 694)**	**Yes (n = 1256)**	***P-*value**	**No (n = 6976)**	**Yes (n = 4974)**	***P-*value**
Age, years, mean (SD)	59.2 (9.6)	58.1 (8.9)	68.0 (10.1)	<0.001	58.6 (9.3)	60.0 (9.9)	<0.001
Male, n (%)	5729 (47.9)	5118 (47.9)	611 (48.6)	0.597	3512 (50.3)	2217 (44.6)	<0.001
Unmarried/others, n (%)	1569 (13.1)	1210 (11.3)	359 (28.6)	<0.001	855 (12.3)	714 (14.4)	<0.001
Educational level, n (%)				<0.001			<0.001
*Illiterate*	3364 (28.2)	2758 (25.8)	606 (48.2)		1853 (26.6)	1511 (30.4)	
*Primary school*	4833 (40.4)	4340 (40.6)	493 (39.3)		2887 (41.4)	1946 (39.1)	
*Middle or high school*	3566 (29.8)	3416 (31.9)	150 (11.9)		2134 (30.6)	1432 (28.8)	
*College or above*	187 (1.6)	180 (1.7)	7 (0.6)		102 (1.5)	85 (1.7)	
Rural residence, n (%)	7521 (62.9)	6693 (62.6)	828 (65.9)	0.021	4424 (63.4)	3097 (62.3)	0.198
BMI, n (%)*				<0.001			<0.001
*Underweight*	820 (6.9)	638 (6.0)	182 (14.5)		430 (6.2)	390 (7.8)	
*Normal*	6219 (52.0)	5530 (51.7)	689 (54.9)		3709 (53.2)	2510 (50.5)	
*Overweight*	3442 (28.8)	3205 (30.0)	237 (18.9)		2006 (28.8)	1436 (28.9)	
*Obese*	1362 (11.4)	1252 (11.7)	110 (8.8)		778 (11.2)	584 (11.7)	
Household income, CNY¥, median (IQR)*	3540.0 (883.3-9213.3)	3800.0 (965.0-9650.0)	1920.0 (533.3-5925.0)	<0.001	3600.0 (930.0-9210.0)	3375.0 (820.0-9216.7)	0.171
Occupation, n (%)*				<0.001			<0.001
*Agricultural work*	4915 (41.1)	4520 (42.3)	395 (31.4)		2995 (42.9)	1920 (38.6)	
*Non-agricultural work*	2682 (22.4)	2596 (24.3)	86 (6.8)		1655 (23.7)	1027 (20.6)	
*Retired*	3932 (32.9)	3235 (30.3)	697 (55.5)		2092 (30.0)	1840 (37.0)	
*Unemployed or never work*	258 (2.2)	211 (2.0)	47 (3.7)		151 (2.2)	107 (2.2)	
Social activities (vs. no), n (%)*	5565 (46.6)	5082 (47.5)	483 (38.5)	<0.001	3255 (46.7)	2310 (46.4)	0.834
Smoking status, n (%)*				0.006			<0.001
*Never smoking*	7134 (59.7)	6398 (59.8)	736 (58.6)		4041 (57.9)	3093 (62.2)	
*Past smoking*	1115 (9.3)	967 (9.0)	148 (11.8)		665 (9.5)	450 (9.0)	
*Current smoking*	3700 (31.0)	3328 (31.1)	372 (29.6)		2270 (32.5)	1430 (28.7)	
Drinking status, n (%)*				<0.001			<0.001
*Never drinking*	7998 (66.9)	7062 (66.0)	936 (74.5)		4497 (64.5)	3501 (70.4)	
*≤1 time/mo*	1100 (9.2)	1005 (9.4)	95 (7.6)		675 (9.7)	425 (8.5)	
*>1 time/mo*	2222 (18.6)	2058 (19.2)	164 (13.1)		1392 (20.0)	830 (16.7)	
Multimorbidity, n (%)*				<0.001			<0.001
*0*	3626 (30.3)	3350 (31.3)	276 (22.0)		2227 (31.9)	1399 (28.1)	
*1*	3423 (28.6)	3074 (28.7)	349 (27.8)		1999 (28.7)	1424 (28.6)	
*≥2*	4631 (38.8)	4028 (37.7)	603 (48.0)		2601 (37.3)	2030 (40.8)	
Visual problems, n (%)*	827 (6.9)	686 (6.4)	141 (11.2)	<0.001	436 (6.3)	391 (7.9)	<0.001
Hearing problems, n (%)*	1108 (9.3)	889 (8.3)	219 (17.5)	<0.001	605 (8.7)	503 (10.1)	0.007
Depression score, mean (SD)*	8.42 (6.32)	8.13 (6.18)	10.91 (6.90)	<0.001	8.14 (6.19)	8.81 (6.47)	<0.001
Cognitive score, mean (SD)*	12.14 (3.41)	12.30 (3.34)	10.34 (3.70)	<0.001	12.27 (3.35)	11.96 (3.50)	<0.001
HGS, kg, mean (SD)	32.58 (10.47)	34.30 (9.50)	17.95 (5.86)	<0.001	33.39 (10.14)	31.45 (10.82)	<0.001
HGS ratio, mean (SD)	0.96 (0.23)	0.96 (0.24)	0.96 (0.21)	0.447	0.98 (0.05)	0.92 (0.35)	<0.001

BMI – body mass index, CNY¥ – Chinese yuan, HGS – handgrip strength, IQR – interquartile range, SD – standard deviation

*Missing data: 107 for BMI, 4290 for household income, 163 for occupation, 126 for social activities, 1 for smoking, 630 for drinking, 270 for multimorbidity, 3 for visual problems, 7 for hearing problems, 174 for depression score, and 2862 for cognitive function.

### Cross-sectional associations of HGS status with functional outcomes

**Figure 2:**
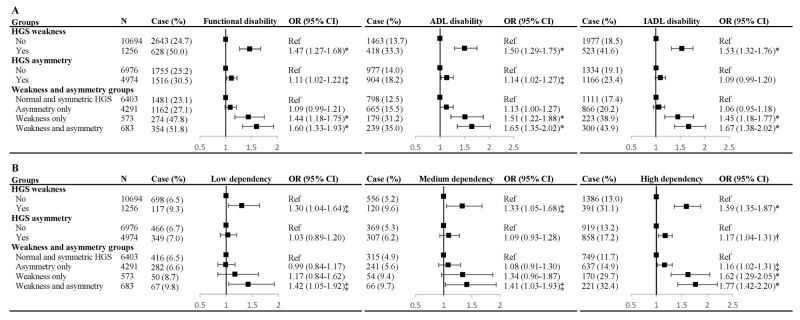
Cross-sectional associations of HGS status with functional outcomes. **Panel A.** Functional disability. **Panel B.** functional dependency. Adjusted for age, sex, marital status, educational level, residence, household income, occupation, social activities, smoking, drinking, multimorbidity, visual and hearing problems, depression score, cognitive function, and BMI. *P < 0.001. †*P* < 0.01. ‡*P* < 0.05. ADL – activities of daily living, CI – confidence interval, HGS – handgrip strength, IADL – instrumental activities of daily living, OR – odds ratio

Among the 11 950 participants, 3271 (27.4%) had functional disability. The prevalence of functional disability was significantly higher in those with HGS weakness or asymmetry (non-weakness vs. weakness = 24.7 vs. 50.0%, symmetry vs. asymmetry = 25.2 vs. 30.5%, **Figure 2**, panel A). After adjustment for covariates, HGS weakness was independently associated with functional disability (OR = 1.47; 95% CI = 1.27–1.68), including ADL disability (OR = 1.50; 95% CI = 1.29–1.75) and IADL disability (OR = 1.53; 95% CI = 1.32–1.76) (**Figure 2**, panel A). HGS asymmetry was also significantly associated with functional disability (OR = 1.11; 95% CI = 1.02–1.22), ADL disability (OR = 1.14; 95% CI = 1.02–1.27), but not IADL disability (OR = 1.09; 95% CI = 0.99–1.20). When comparing with those with normal and symmetric HGS, participants combined with weakness and asymmetry had the greatest odds for functional disability (OR = 1.60; 95% CI = 1.33–1.93), ADL disability (OR = 1.65; 95% CI = 1.35–2.02), and IADL disability (OR = 1.67; 95% CI = 1.38–2.02). The cross-sectional associations between HGS measures and functional disability were unchanged in the complete data sets (Table S5 in the **Online Supplementary Document**). Similar results were noted when HGS asymmetry was defined by the 20 and 30% rule (Table S6 in the **Online Supplementary Document**). Analyses treating the outcome measures as continuous variables found that participants with weakness and asymmetry together scored significantly higher in functional disability (Table S7 in the **Online Supplementary Document**).

The associations between HGS status and functional dependency were displayed in **Figure 2**, panel B. The presence of either weakness (OR = 1.59; 95% CI = 1.35–1.87) or asymmetry (OR = 1.17; 95% CI = 1.04–1.31) was associated with an increased prevalence of high dependency. Relative to participants with normal and symmetric HGS, those with both weakness and asymmetry were more likely to suffer from functional dependency, including low dependency (OR = 1.42; 95% CI = 1.05–1.92), medium dependency (OR = 1.41; 95% CI = 1.03–1.93), and high dependency (OR = 1.77; 95% CI = 1.42–2.20). The complete case analysis confirmed the weakness and asymmetry in association with high functional dependency (Table S8 in the **Online Supplementary Document**). Analyses using different asymmetry cutoffs yielded similar relationships between HGS status and functional dependency (Table S9 in the **Online Supplementary Document**).

### Longitudinal associations of HGS status with functional outcomes

During the four-year follow-up (average follow-up time 47.7 months), 1822 (24.2%) participants developed functional disability. Compared with individuals with normal HGS, participants with HGS weakness were more likely to experience new-onset functional disability (OR = 1.59; 95% CI = 1.30–1.95), including ADL disability (OR = 1.49; 95% CI = 1.19–1.86) and IADL disability (OR = 1.50; 95% CI = 1.21–1.86) (**Table 2**). Subjects with HGS asymmetry also had higher risk of functional disability (OR = 1.18; 95% CI = 1.05–1.32), ADL disability (OR = 1.17; 95% CI = 1.02–1.34), and IADL disability (OR = 1.18; 95% CI = 1.04–1.35) than those with HGS symmetry. In comparison with those with normal and symmetric HGS, respondents combined with weakness and asymmetry had the greatest risk of incident functional disability (OR = 1.91; 95% CI = 1.45–2.52), followed by those with weakness only (OR = 1.47; 95% CI = 1.10–1.96) and those with asymmetry only (OR = 1.15; 95% CI = 1.02–1.29). Participants with both weakness and asymmetry also had the highest risk of ADL disability (OR = 1.98; 95% CI = 1.48–2.65) and IADL disability (OR = 1.76; 95% CI = 1.32–2.35). The relationships were not significantly changed among the subpopulations of 3452 subjects with complete data (Table S10 in the **Online Supplementary Document**). The results remained similar with the HGS ratio at 20% (Table S11 in the **Online Supplementary Document**). When the HGS ratio at 30%, weakness and asymmetry together predicted ADL disability (OR = 2.28; 95% CI = 1.23–4.23), but not IADL disability (OR = 1.23; 95% CI = 0.64–2.34). Linear models revealed that both weakness and asymmetry were associated with higher disability scores (Table S12 in the **Online Supplementary Document**).

**Table 2:** Odds ratio (95% CI) for the longitudinal association of HGS status with functional disability

Groups	HGS weakness		HGS asymmetry		Weakness and asymmetry combined groups			
	**No**	**Yes**	**No**	**Yes**	**Normal and symmetric HGS**	**Asymmetry only**	**Weakness only**	**Weakness and asymmetry**
**Functional disability**								
Case/Number	1601/7035	221/505	1016/4556	806/2984	919/4313	682/2722	97/243	124/262
Model 1	Ref	2.66 (2.21–3.20)*	Ref	1.29 (1.16–1.43)*	Ref	1.24 (1.10–1.38)*	2.49 (1.91–3.26)*	3.31 (2.56–4.26)*
Model 2	Ref	1.96 (1.61–2.38)*	Ref	1.20 (1.07–1.34)*	Ref	1.16 (1.04–1.31)†	1.86 (1.41–2.45)*	2.32 (1.78–3.02)*
Model 3	Ref	1.59 (1.30–1.95)*	Ref	1.18 (1.05–1.32)†	Ref	1.15 (1.02–1.29)‡	1.47 (1.10–1.96)†	1.91 (1.45–2.52)*
**ADL disability**								
Case/Number	972/7035	144/505	615/4556	501/2984	560/4313	412/2722	55/243	89/262
Model 1	Ref	2.49 (2.03–3.06)*	Ref	1.29 (1.14–1.47)*	Ref	1.19 (1.04–1.37)‡	1.97 (1.44–2.70)*	3.43 (2.62–4.50)*
Model 2	Ref	1.82 (1.47–2.26)*	Ref	1.20 (1.05–1.37)†	Ref	1.13 (0.98–1.30)	1.45 (1.05–2.00)‡	2.41 (1.82–3.19)*
Model 3	Ref	1.49 (1.19–1.86)*	Ref	1.17 (1.02–1.34)‡	Ref	1.10 (0.96–1.27)	1.15 (0.83–1.61)	1.98 (1.48–2.65)*
**IADL disability**								
Case/Number	1137/7035	166/505	716/4556	587/2984	643/4313	494/2722	73/243	93/262
Model 1	Ref	2.56 (2.10–3.11)*	Ref	1.31 (1.17–1.48)*	Ref	1.27 (1.12–1.44)*	2.49 (1.87–3.32)*	3.14 (2.40–4.10)*
Model 2	Ref	1.85 (1.51–2.28)*	Ref	1.21 (1.07–1.37)†	Ref	1.19 (1.04–1.36)†	1.83 (1.36–2.46)*	2.15 (1.63–2.84)*
Model 3	Ref	1.50 (1.21–1.86)*	Ref	1.18 (1.04–1.35)‡	Ref	1.16 (1.01–1.33)‡	1.43 (1.05–1.94)‡	1.76 (1.32–2.35)*

ADL – activities of daily living, BMI – body mass index, CI – confidence interval, HGS – handgrip strength, IADL – instrumental activities of daily living

Model 1: crude model; Model 2: adjusted for age and sex; Model 3: adjusted as model 2 plus marital status, educational level, residence, household income, occupation, social activities, smoking, drinking, multimorbidity, visual and hearing problems, depression score, cognitive function, BMI, and follow-up time.

*P < 0.001.

†*P* < 0.01.

‡*P* < 0.05.

For functional dependency, we found that exposure to weakness (OR = 1.67; 95% CI = 1.32–2.11) or asymmetry (OR = 1.20; 95% CI = 1.04–1.38) was significantly associated with high dependency (**Table 3**). The risk of high dependency further increased if participants combined with weakness and asymmetry (OR = 2.17; 95% CI = 1.59–2.97). Similar results were observed by analyses with complete data sets (Table S13 in the **Online Supplementary Document**) and analyses using different asymmetry cutoffs (Table S14 in the **Online Supplementary Document**).

**Table 3:** Multinominal logistic regression for the longitudinal association of HGS status with functional dependency

Groups	Low dependency		Medium dependency		High dependency	
	**Case/Number**	OR (95% CI)*	**Case/Number**	OR (95% CI)*	**Case/Number**	OR (95% CI)*
HGS weakness						
*No*	453/7035	Ref	236/7035	Ref	901/7035	Ref
*Yes*	46/505	1.36 (0.96–1.91)	35/505	1.66 (1.11–2.47)§	137/505	1.67 (1.32–2.11)†
HGS asymmetry						
*No*	295/4556	Ref	139/4556	Ref	573/4556	Ref
*Yes*	204/2984	1.05 (0.86–1.27)	132/2984	1.38 (1.08–1.78)§	465/2984	1.20 (1.04–1.38)§
Weakness and asymmetry combined groups						
*Normal and symmetric HGS*	272/4313	Ref	119/4313	Ref	520/4313	Ref
*Asymmetry only*	181/2722	1.04 (0.85–1.27)	117/2722	1.49 (1.14–1.94)‡	381/2722	1.13 (0.97–1.31)
*Weakness only*	23/243	1.34 (0.83–2.16)	20/243	2.24 (1.33–3.79)‡	53/243	1.35 (0.95–1.91)
*Weakness and asymmetry*	23/262	1.41 (0.87–2.26)	15/262	1.73 (0.96–3.12)	84/262	2.17 (1.59–2.97)†

CI – confidence interval, HGS – handgrip strength, OR – odds ratio, Ref – reference

*Adjusted for age, sex, marital status, educational level, residence, household income, occupation, social activities, smoking, drinking, multimorbidity, visual and hearing problems, depression score, cognitive function, BMI, and follow-up time.

†*P* < 0.001.

‡*P* < 0.01.

§*P* < 0.05.

### Subgroup analyses

Figure S1 in the **Online Supplementary Document** presented the results of subgroup analyses. The associations of weakness and asymmetry with incident functional disability were not changed by age, sex, and hand dominance (all *P* for interaction >0.05). Similarly, subgroup analyses revealed that age, sex, and hand dominance did not significantly modify the relationships between HGS status and functional dependency (all *P* for interaction >0.05, Figure S2 in the **Online Supplementary Document**).

## DISCUSSION

The present study found that HGS asymmetry and weakness were independently associated with functional disability and functional dependency among middle-aged and older Chinese adults. HGS asymmetry and weakness affected functional health additively, with participants with both weakness and asymmetry having the greatest risk of functional disability and high dependency.

Our results were compatible with previous studies reporting that declines in HGS predicted ADL/IADL disability and loss of independence [9,10,23]. Several explanations may account for this finding. First, HGS is a marker of physical activity, which itself holds important functions [11]. Decreased HGS and hand dexterity may directly affect the function of the upper limbs, impairing the ability to complete self-care activities (e.g. eating, dressing, cooking, shopping) [9,41]. Besides, lower HGS was also related to poor physical performance in the lower limbs, such as slow gait speed and mobility impairment [11,12,42], which may further exacerbate functional decline. Second, HGS weakness may reflect reduced neurological function or brain health [43], though it is primarily regarded as age-related deficits in the musculoskeletal system [43]. The measurement of HGS requires complex coordination movements that are heavily regulated by the neural systems [43]. It has been reported that higher HGS was associated with larger brain volumes in areas relevant to executive and memory function [44]. Adults with reduced HGS may experience an accelerated decline in neurocognitive function [19,20] and have a higher risk of future stroke [45]. The dysfunction of the nervous system may reduce the coordination of various tasks, such as difficulty in getting things, taking more time to finish tasks, and asymmetric motor performance [17,46], limiting the capacity to execute daily living activities and causing the resultant disability. Third, HGS weakness may place older adults at greater risk for metabolic dysfunction and chronic morbidities (e.g. insulin resistance, diabetes, and cardiovascular disease) [12,42]. The presence and accumulation of these chronic diseases may potentiate the disabling process [47].

Consistent with prior studies from Aging Americans [21–23], we found that Chinese adults with HGS asymmetry had an elevated risk of functional disability. Though our results suggest that HGS weakness is more robustly related to functional disability, HGS asymmetry may pose similar health consequences that aggravate the disability risk. Humans prefer to use their dominant hands in daily activities [48], and the overreliance on the dominant hand while underuse of the nondominant hand may exacerbate strength imbalance. Other factors such as acute and chronic injuries may also affect hand usage and strength, causing asymmetry in HGS. A large difference in bilateral HGS may represent asymmetric motor function, which may factor into physical decline and functional limitations [22,23]. Indeed, previous studies have found that the presence of HGS asymmetry was related to poorer standing balance and timed-up-and-go performance [49,50], and could predict future limitations in activities like transferring and toileting [22]. Moreover, HGS asymmetry may indicate an imbalance in brain hemisphere activation or impaired neural system functioning [20,22,48]. Growing evidence has suggested that HGS asymmetry would increase the risk of neurodegenerative disorders [17], motoric cognitive risk syndrome [19], and cognitive impairment [20]. The cognitive vulnerability may further exacerbate the functional loss [51], and prior studies have demonstrated that individuals with HGS asymmetry had greater odds for limitations in tasks that require higher neuropsychological functioning (e.g. managing money and using maps) [23]. Our results may be supported by these findings. However, the mechanisms underlying the relationship of HGS asymmetry and weakness with functional disability are not completely understood and await further investigation.

The combination of HGS asymmetry and weakness showed a stronger association with functional disability than either of them alone in our study. Although the additive effects of HGS asymmetry and weakness on health outcomes (e.g. cognitive function [20], morbidity accumulation [18], and mortality [52]) have been reported in recent studies, to our knowledge, only two studies have examined their joint association with functional disability [15,21]. A cross-sectional study found that older Americans with HGS asymmetry and weakness had greater odds for limitations in ADL and IADL activities [15]. Similarly, a longitudinal cohort from the Health and Retirement Study revealed that the presence of both HGS asymmetry and weakness was more strongly related to future ADL disability [21]. These findings collectively suggest that functional decline may be accelerated in those with concurrent HGS asymmetry and weakness. Our study supports and complements the existing literature, addressing the gap in previous research which was limited by the lack of epidemiological studies on this topic in developing regions like China.

Our findings have important clinical implications. China is currently experiencing rapid population aging accompanied by a rising burden of functional impairment, which often contributes to an increased risk for disability, reduced quality of life, and socioeconomic burden [2]. Understanding the determinants of functional disability, especially in middle age and old age, is imperative to develop effective countermeasures and guide appropriate public health initiatives. Our study suggests that the presence of HGS asymmetry and weakness may potentiate functional decline. HGS asymmetry, a simple and quick measure, should be evaluated in clinical and research settings as an addition to HGS measurement protocols. The combined assessment of HGS asymmetry with weakness may help to better identify at-risk populations for functional disability. Interventions to improve muscle strength and correct strength asymmetry (e.g. exercise training programmes, nutrition therapy, task-oriented motor learning and related bilateral and unilateral therapeutic physical activities) [53,54] may prevent or delay functional disability and improve quality of life for middle-aged and older adults.

The present study has several advantages. First, this is the first study to examine the individual and combined effects of HGS asymmetry and weakness on functional disability in the Chinese population based on the cross-sectional and prospective analysis. Second, we employed a nationally representative longitudinal survey, and the large sample size made our results reliable. Third, functional status was measured not only by binary disability variables but also by continuous functional scores. Besides, the disability burden was further evaluated through the level of dependency reflected in the interval of care needs, which may provide a clearer indication of the intensity of care required than usual disability measures.

### Limitations

Some limitations must be noted. First, we excluded ambidextrous participants and those lacking HGS data on either hand, limiting the generalisability of our results to the general middle-aged and older Chinese population. Second, selection bias may be introduced, as we eliminated participants without hand dominance or HGS data and those who had missing functional status during the two waves. Individuals who cannot complete the HGS test and those who lost to follow-up or died after their baseline interview may have experienced an accelerated decline in their strength and functional ability. The exclusion of these participants may underestimate the actual association between HGS measures and functional disability. Caution thus should be taken when interpreting and extrapolating our findings. Further well-designed, large-sample studies are warranted to verify our results. Third, self-reported measures (e.g. ADL/IADL and hand dominance) may cause potential recall bias, even though this method has been widely used in population-based studies [17,21,32,33]. Fourthly, HGS differences between hands may vary among individuals [14]. Although we defined HGS asymmetry by three different rules (i.e. 10, 20, and 30%), future studies are needed to create a robust HGS asymmetry cut-off point. Fifthly, regarding drinking status, as data on the quantity of alcohol consumption was unavailable in CHARLS, we were unable to assess the potential effect of drinking amount on functional disability, which needs further research. Lastly, despite that we have adjusted for multiple potential variables, residual confounding from unmeasured factors may still exist, for example, dietary intake and other time-varying confounding. More research is needed to validate our results and confirm the clinical applicability of HGS asymmetry.

## CONCLUSIONS

HGS asymmetry and weakness were associated with higher risks of functional disability and functional dependency among middle-aged and older Chinese adults. Our findings extend the knowledge regarding associations between HGS measures and functional health, and suggest that assessment of HGS asymmetry together with the maximal HGS may help identify individuals at risk of functional disability. Interventions of HGS asymmetry and weakness may prevent the disabling process.

## Additional material


Online Supplementary Document
