# Supplementary material for: Association of handgrip strength asymmetry and weakness with functional disability among middle-aged and older adults in China
Source: J Glob Health. 2024 Mar 29;14:04047. doi: 10.7189/jogh.14.04047 (PMC10979250; doi:10.7189/jogh.14.04047)

Association of handgrip strength asymmetry and weakness with functional disability among middle-aged and older adults in China

Online Supplementary Documents

Table S1 Functional dependency by interval-of-need dependency categorization.

Table S2 Baseline characteristics of 11950 participants by the combined HGS weakness and asymmetry categories in the cross-sectional analysis.

Table S3 Baseline characteristics of 7540 participants without functional disability by HGS status in the prospective analysis.

Table S4 Baseline characteristics of 7540 participants without functional disability by the combined HGS weakness and asymmetry categories in the prospective analysis.

Table S5 Cross-sectional association between HGS status and functional disability in subpopulations of 5288 participants with complete data.

Table S6 Cross-sectional association between HGS status and functional disability, with HGS asymmetry defined by 20% and 30% rule.

Table S7 Cross-sectional association between HGS status and disability scores.

Table S8 Cross-sectional association between HGS status and functional dependency in subpopulations of 5288 participants with complete data.

Table S9 Cross-sectional association between HGS status and functional dependency, with HGS asymmetry defined by 20% and 30% rule.

Table S10 Longitudinal association of HGS status with functional disability in subpopulations of 3452 participants with complete data.

Table S11 Longitudinal association between HGS status and functional disability, with HGS asymmetry defined by 20% and 30% rule.

Table S12 Longitudinal association of HGS status with disability scores.

Table S13 Longitudinal association between HGS status and functional dependency in subpopulations of 3452 participants with complete data.

Table S14 Longitudinal association between HGS status and functional dependency, with HGS asymmetry defined by 20% and 30% rule.

Figure S1 Subgroup analyses for the longitudinal associations of HGS status with functional disability.

Figure S2 Subgroup analyses for the longitudinal association of HGS status with functional dependency.

Table S1. Functional dependency by interval-of-need dependency categorization

Categories	Definition
High dependency	Difficulty in eating, dressing, toileting, getting in or out of bed, or controlling urination and defecation
Medium dependency	Difficulty in cooking or taking medications, and no difficulty in the items defined in the high dependency
Low dependency	Difficulty in bathing, shopping, managing money, or doing housework, and no difficulty in the items defined in medium and high dependency
Independent	No difficulty with the above items

Table S2. Baseline characteristics of 11950 participants by the combined HGS weakness and asymmetry categories in the cross-sectional analysis

**Characteristics**	**Normal and symmetric HGS (n=6403)**	**Asymmetry only (n=4291)**	**Weakness only (n=573)**	**Weakness and asymmetry (n=683)**	***P***
Age, years, mean (SD)	57.7 (8.7)	58.7 (9.2)	67.9 (9.9)	68.02 (10.2)	<0.001
Male, n (%)	3213 (50.2)	1905 (44.4)	299 (52.2)	312 (45.7)	<0.001
Unmarried/others, n (%)	692 (10.8)	518 (12.1)	163 (28.4)	196 (28.7)	<0.001
Educational level, n (%)					<0.001
Illiterate	1585 (24.8)	1173 (27.3)	268 (46.8)	338 (49.5)	
Primary school	2644 (41.3)	1696 (39.5)	243 (42.4)	250 (36.6)	
Middle or high school	2072 (32.4)	1344 (31.3)	62 (10.8)	88 (12.9)	
College or above	102 (1.6)	78 (1.8)	0 (0.0)	7 (1.0)	
Rural residence, n (%)	4035 (63.0)	2658 (61.9)	389 (67.9)	439 (64.3)	0.039
BMI, n (%)*					<0.001
Underweight	348 (5.4)	290 (6.8)	82 (14.3)	100 (14.6)	
Normal	3382 (52.8)	2148 (50.1)	327 (57.1)	362 (53.0)	
Overweight	1908 (29.8)	1297 (30.2)	98 (17.1)	139 (20.4)	
Obese	728 (11.4)	524 (12.2)	50 (8.7)	60 (8.8)	
Household income, CNY¥, median (IQR)*	3868.3 (1000.0-9675.0)	3672.5 (902.5-9600.0)	1803.5 (435.0-4895.0)	1950.0 (600.0-6900.0)	<0.001
Occupation, n (%)*					<0.001
Agricultural work	2777 (43.4)	1743 (40.6)	218 (38.0)	177 (25.9)	
Non-agricultural work	1613 (25.2)	983 (22.9)	42 (7.3)	44 (6.4)	
Retired	1813 (28.3)	1422 (33.1)	279 (48.7)	418 (61.2)	
Unemployed or never work	130 (2.0)	81 (1.9)	21 (3.7)	26 (3.8)	
Social activities (vs no), n (%)*	3033 (47.4)	2049 (47.8)	222 (38.7)	261 (38.2)	<0.001
Smoking status, n (%)*					<0.001
Never smoking	3717 (58.1)	2681 (62.5)	324 (56.5)	412 (60.3)	
Past smoking	594 (9.3)	373 (8.7)	71 (12.4)	77 (11.3)	
Current smoking	2092 (32.7)	1236 (28.8)	178 (31.1)	194 (28.4)	
Drinking status, n (%)*					<0.001

Never drinking	4087 (63.8)	2975 (69.3)	410 (71.6)	526 (77.0)	
≤1 time/month	630 (9.8)	375 (8.7)	45 (7.9)	50 (7.3)	
>1 time/month	1313 (20.5)	745 (17.4)	79 (13.8)	85 (12.4)	
Multimorbidity, n (%)*					<0.001
0	2090 (32.6)	1260 (29.4)	137 (23.9)	139 (20.4)	
1	1846 (28.8)	1228 (28.6)	153 (26.7)	196 (28.7)	
≥2	2330 (36.4)	1698 (39.6)	271 (47.3)	332 (48.6)	
Visual problems, n (%)*	369 (5.8)	317 (7.4)	67 (11.7)	74 (10.9)	<0.001
Hearing problems, n (%)*	507 (7.9)	382 (8.9)	98 (17.1)	121 (17.8)	<0.001
Depression score, mean (SD)*	7.92 (6.10)	8.44 (6.29)	10.61 (6.63)	11.16 (7.11)	<0.001
Cognitive score, mean (SD)*	12.39 (3.30)	12.15 (3.41)	10.41 (3.61)	10.28 (3.79)	<0.001
HGS, kg, mean (SD)	34.69 (9.40)	33.72 (9.62)	18.82 (5.58)	17.22 (5.99)	<0.001
HGS ratio, mean (SD)	0.98 (0.05)	0.92 (0.37)	0.99 (0.05)	0.94 (0.28)	<0.001

HGS – handgrip strength, BMI, body mass index, SD – standard deviation, IQR – interquartile range.

*Missing data: 107 for BMI, 4290 for household income, 163 for occupation, 126 for social activities, 1 for smoking, 630 for drinking, 270 for multimorbidity, 3 for visual problems, 7 for hearing problems, 174 for depression score, and 2862 for cognitive function.

Table S3. Baseline characteristics of 7540 participants without functional disability by HGS status in the prospective analysis

**Characteristics**	**Total (n=7540)**	**HGS weakness**			**HGS asymmetry**		
		**No (n=7035)**	**Yes (n=505)**	***P***	**No (n=4556)**	**Yes (n=2984)**	***P***
Age, years, mean (SD)	57.5 (8.6)	57.0 (8.4)	64.6 (9.1)	<0.001	57.1 (8.4)	58.1 (9.0)	<0.001
Male, n (%)	3779 (50.1)	3535 (50.2)	244 (48.3)	0.402	2384 (52.3)	1395 (46.7)	<0.001
Unmarried/others, n (%)	770 (10.2)	665 (9.5)	105 (20.8)	<0.001	451 (9.9)	319 (10.7)	0.267
Educational level, n (%)				<0.001			0.010
Illiterate	1768 (23.4)	1567 (22.3)	201 (39.8)		1012 (22.2)	756 (25.3)	
Primary school	3057 (40.5)	2839 (40.4)	218 (43.2)		1895 (41.6)	1162 (38.9)	
Middle or high school	2606 (34.6)	2520 (35.8)	86 (17.0)		1587 (34.8)	1019 (34.1)	
College or above	109 (1.4)	109 (1.5)	0 (0.0)		62 (1.4)	47 (1.6)	
Rural residence, n (%)	4689 (62.2)	4371 (62.1)	318 (63.0)	0.708	2856 (62.7)	1833 (61.4)	0.270
BMI, n (%)*				<0.001			0.005
Underweight	423 (5.6)	364 (5.2)	59 (11.7)		226 (5.0)	197 (6.6)	
Normal	3974 (52.7)	3693 (52.5)	281 (55.6)		2453 (53.8)	1521 (51.0)	
Overweight	2218 (29.4)	2110 (30.0)	108 (21.4)		1339 (29.4)	879 (29.5)	
Obese	900 (11.9)	847 (12.0)	53 (10.5)		526 (11.5)	374 (12.5)	
Household income, CNY¥, median (IQR)*	4100.0 (1087.5-10033.3)	4316.7 (1160.0-10220.0)	2200.0 (600.0-6370.0)	<0.001	4200.0 (1159.0-10050.0)	4000.0 (1000.0-10020.0)	0.343
Occupation, n (%)*				<0.001			<0.001
Agricultural work	3287 (43.6)	3076 (43.7)	211 (41.8)		2041 (44.8)	1246 (41.8)	
Non-agricultural work	1988 (26.4)	1942 (27.6)	46 (9.1)		1237 (27.2)	751 (25.2)	
Retired	2041 (27.1)	1817 (25.8)	224 (44.4)		1154 (25.3)	887 (29.7)	
Unemployed or never work	142 (1.9)	127 (1.8)	15 (3.0)		81 (1.8)	61 (2.0)	
Social activities (vs no), n (%)*	3743 (49.6)	3524 (50.1)	219 (43.4)	0.004	2259 (49.6)	1484 (49.7)	0.941
Smoking status, n (%)*				0.707			0.002
Never smoking	4469 (59.3)	4167 (59.2)	302 (59.8)		2629 (57.7)	1840 (61.7)	
Past smoking	614 (8.1)	569 (8.1)	45 (8.9)		377 (8.3)	237 (7.9)	
Current smoking	2456 (32.6)	2298 (32.7)	158 (31.3)		1550 (34.0)	906 (30.4)	
Drinking status, n (%)*				<0.001			0.002
Never drinking	4894 (64.9)	4524 (64.3)	370 (73.3)		2874 (63.1)	2020 (67.7)	

≤1 time/month	729 (9.7)	695 (9.9)	34 (6.7)		456 (10.0)	273 (9.1)	
>1 time/month	1522 (20.2)	1440 (20.5)	82 (16.2)		966 (21.2)	556 (18.6)	
Multimorbidity, n (%)*				<0.001			0.009
0	2640 (35.0)	2496 (35.5)	144 (28.5)		1653 (36.3)	987 (33.1)	
1	2254 (29.9)	2109 (30.0)	145 (28.7)		1356 (29.8)	898 (30.1)	
≥2	2481 (32.9)	2275 (32.3)	206 (40.8)		1450 (31.8)	1031 (34.6)	
Visual problems, n (%)*	369 (4.9)	337 (4.8)	32 (6.3)	0.120	208 (4.6)	161 (5.4)	0.103
Hearing problems, n (%)*	464 (6.2)	411 (5.8)	53 (10.5)	<0.001	274 (6.0)	190 (6.4)	0.539
Depression score, mean (SD)*	7.21 (5.62)	7.06 (5.53)	9.28 (6.38)	<0.001	7.06 (5.52)	7.43 (5.77)	0.005
Cognitive score, mean (SD)*	12.53 (3.25)	12.62 (3.19)	10.98 (3.77)	<0.001	12.62 (3.20)	12.38 (3.32)	0.004
HGS, kg, mean (SD)	34.07 (10.19)	35.19 (9.51)	18.58 (5.84)	<0.001	34.58 (9.91)	33.30 (10.57)	<0.001
HGS ratio, mean (SD)	0.96 (0.26)	0.96 (0.27)	0.96 (0.18)	0.858	0.98 (0.05)	0.92 (0.41)	<0.001

HGS – handgrip strength, BMI – body mass index, SD – standard deviation, IQR – interquartile range.

*Missing data: 25 for BMI, 2902 for household income, 82 for occupation, 36 for social activities, 1 for smoking, 395 for drinking, 165 for multimorbidity, 1 for visual problems, 4 for hearing problems, 57 for depression score, and 1449 for cognitive function.

Table S4. Baseline characteristics of 7540 participants without functional disability by the combined HGS weakness and asymmetry categories in the prospective analysis

**Characteristics**	**Normal and symmetric HGS (n=4313)**	**Asymmetry only (n=2722)**	**Weakness only (n=243)**	**Weakness and asymmetry (n=262)**	***P***
Age, years, mean (SD)	56.7 (8.2)	57.4 (8.7)	64.2 (8.7)	64.9 (9.3)	<0.001
Male, n (%)	2258 (52.4)	1277 (46.9)	126 (51.9)	118 (45.0)	<0.001
Unmarried/others, n (%)	405 (9.4)	260 (9.6)	46 (18.9)	59 (22.5)	<0.001
Educational level, n (%)					<0.001
Illiterate	918 (21.3)	649 (23.8)	94 (38.7)	107 (40.8)	
Primary school	1787 (41.4)	1052 (38.6)	108 (44.4)	110 (42.0)	
Middle or high school	1546 (35.8)	974 (35.8)	41 (16.9)	45 (17.2)	
College or above	62 (1.4)	47 (1.7)	0 (0.0)	0 (0.0)	
Rural residence, n (%)	2696 (62.5)	1675 (61.5)	160 (65.8)	158 (60.3)	0.483
BMI, n (%)*					<0.001
Underweight	196 (4.5)	168 (6.2)	30 (12.3)	29 (11.1)	
Normal	2308 (53.5)	1385 (50.9)	145 (59.7)	136 (51.9)	
Overweight	1293 (30.0)	817 (30.0)	46 (18.9)	62 (23.7)	
Obese	506 (11.7)	341 (12.5)	20 (8.2)	33 (12.6)	
Household income, CNY¥, median (IQR) *	4450.0 (1200.0-10275.3)	4100.0 (1077.1-10135.0)	2143.5 (660.0-5675.0)	2262.0 (600.0-7550.0)	<0.001
Occupation, n (%)*					<0.001
Agricultural work	1921 (44.5)	1155 (42.4)	120 (49.4)	91 (34.7)	
Non-agricultural work	1217 (28.2)	725 (26.6)	20 (8.2)	26 (9.9)	
Retired	1059 (24.6)	758 (27.8)	95 (39.1)	129 (49.2)	
Unemployed or never work	77 (1.8)	50 (1.8)	4 (1.6)	11 (4.2)	
Social activities (vs no), n (%)*	2157 (50.0)	1367 (50.2)	102 (42.0)	117 (44.7)	0.033
Smoking status, n (%)*					0.032
Never smoking	2490 (57.7)	1677 (61.6)	139 (57.2)	163 (62.2)	
Past smoking	357 (8.3)	212 (7.8)	20 (8.2)	25 (9.5)	
Current smoking	1466 (34.0)	832 (30.6)	84 (34.6)	74 (28.2)	

Drinking status, n (%)*					<0.001
Never drinking	2704 (62.7)	1820 (66.9)	170 (70.0)	200 (76.3)	
≤1 time/month	440 (10.2)	255 (9.4)	16 (6.6)	18 (6.9)	
>1 time/month	923 (21.4)	517 (19.0)	43 (17.7)	39 (14.9)	
Multimorbidity, n (%)*					<0.001
0	1582 (36.7)	914 (33.6)	71 (29.2)	73 (27.9)	
1	1288 (29.9)	821 (30.2)	68 (28.0)	77 (29.4)	
≥2	1351 (31.3)	924 (33.9)	99 (40.7)	107 (40.8)	
Visual problems, n (%)*	189 (4.4)	148 (5.4)	19 (7.8)	13 (5.0)	0.035
Hearing problems, n (%)*	246 (5.7)	165 (6.1)	28 (11.5)	25 (9.5)	<0.001
Depression score, mean (SD)*	6.95 (5.47)	7.24 (5.64)	9.05 (6.07)	9.48 (6.65)	<0.001
Cognitive score, mean (SD)*	12.70 (3.15)	12.49 (3.24)	11.04 (3.68)	10.92 (3.87)	<0.001
HGS, kg, mean (SD)	35.43 (9.39)	34.80 (9.68)	19.40 (5.73)	17.82 (5.85)	<0.001
HGS ratio, mean (SD)	0.98 (0.05)	0.92 (0.43)	0.98 (0.05)	0.94 (0.24)	<0.001

HGS – handgrip strength, BMI – body mass index, SD – standard deviation, IQR – interquartile range.

*Missing data: 25 for BMI, 2902 for household income, 82 for occupation, 36 for social activities, 1 for smoking, 395 for drinking, 165 for multimorbidity, 1 for visual problems, 4 for hearing problems, 57 for depression score, and 1449 for cognitive function.

Table S5. Cross-sectional association between HGS status and functional disability in subpopulations of 5288 participants with complete data

**Groups**	**Functional disability**			**ADL disability**			**IADL disability**		
	**Model 1 OR (95% CI)**	**Model 2 OR (95% CI)**	**Model 3 OR (95% CI)**	**Model 1 OR (95% CI)**	**Model 2 OR (95% CI)**	**Model 3 OR (95% CI)**	**Model 1 OR (95% CI)**	**Model 2 OR (95% CI)**	**Model 3 OR (95% CI)**
HGS weakness									
No	Ref	Ref	Ref	Ref	Ref	Ref	Ref	Ref	Ref
Yes	3.05 (2.70-3.43)*	1.97 (1.73-2.24)*	1.36 (1.10-1.68)†	3.15 (2.76-3.58)*	1.99 (1.73-2.29)*	1.37 (1.07-1.74)‡	3.15 (2.78-3.56)*	2.07 (1.82-2.37)*	1.48 (1.19-1.86)*
HGS asymmetry									
No	Ref	Ref	Ref	Ref	Ref	Ref	Ref	Ref	Ref
Yes	1.30 (1.20-1.41)*	1.19 (1.09-1.29)*	1.18 (1.03-1.35)‡	1.36 (1.24-1.51)*	1.24 (1.12-1.37)*	1.27 (1.08-1.50)†	1.30 (1.19-1.42)*	1.17 (1.07-1.28)*	1.08 (0.93-1.25)
Weakness and asymmetry combined groups									
Normal and symmetric HGS	Ref	Ref	Ref	Ref	Ref	Ref	Ref	Ref	Ref
Asymmetry only	1.23 (1.13-1.35)*	1.15 (1.05-1.26)†	1.13 (0.98-1.32)	1.29 (1.15-1.44)*	1.20 (1.07-1.35)†	1.21 (1.01-1.45)‡	1.20 (1.09-1.33)*	1.12 (1.01-1.24) ‡	1.03 (0.87-1.21)
Weakness only	3.05 (2.56-3.62)*	1.94 (1.62-2.33)*	1.22 (0.89-1.66)	3.19 (2.63-3.86)*	1.99 (1.62-2.43)*	1.18 (0.82-1.69)	3.03 (2.54-3.63)*	1.97 (1.63-2.39)*	1.33 (0.95-1.84)
Weakness and asymmetry	3.58 (3.04-4.20)*	2.22 (1.88-2.64)*	1.65 (1.24-4.20)*	3.78 (3.18-4.50)*	2.31 (1.92-2.77)*	1.78 (1.30-2.43)*	3.73 (3.17-4.40)*	2.36 (1.99-2.81)*	1.66 (1.24-2.22)*

HGS – handgrip strength, ADL – activities of daily living, IADL – instrumental activities of daily living, OR – odds ratio, CI – confidence interval.

Model 1: crude model; Model 2: adjusted for age and sex; Model 3: adjusted as model 2 plus marital status, educational level, residence, household income, occupation, social activities, smoking, drinking, multimorbidity, visual and hearing problems, depression score, cognitive function, and BMI.

**P*<0.001.

†*P*<0.01.

‡*P*<0.05.

Table S6 Cross-sectional association between HGS status and functional disability, with HGS asymmetry defined by 20% and 30% rule

Groups	Functional disability			ADL disability			IADL disability		
	**Model 1 OR (95% CI)**	**Model 2 OR (95% CI)**	**Model 3 OR (95% CI)**	**Model 1 OR (95% CI)**	**Model 2 OR (95% CI)**	**Model 3 OR (95% CI)**	**Model 1 OR (95% CI)**	**Model 2 OR (95% CI)**	**Model 3 OR (95% CI)**
**HGS asymmetry ratio at 20%**									
HGS asymmetry									
No	Ref	Ref	Ref	Ref	Ref	Ref	Ref	Ref	Ref
Yes	1.64 (1.47-1.84)*	1.41 (1.25-1.59)*	1.27 (1.12-1.45)*	1.67 (1.46-1.90)*	1.41 (1.23-1.62)*	1.25 (1.07-1.45)†	1.73 (1.53-1.96)*	1.48 (1.30-1.68)*	1.33 (1.16-1.52)*
Weakness and asymmetry combined groups									
Normal and symmetric HGS	Ref	Ref	Ref	Ref	Ref	Ref	Ref	Ref	Ref
Asymmetry only	1.49 (1.30-1.70)*	1.35 (1.18-1.55)*	1.26 (1.09-1.46)†	1.53 (1.30-1.79)*	1.38 (1.17-1.63)*	1.28 (1.07-1.52)†	1.52 (1.32-1.75)*	1.36 (1.18-1.59)*	1.26 (1.08-1.48)†
Weakness only	3.00 (2.62-3.44)*	1.95 (1.69-2.25)*	1.47 (1.25-1.72)*	3.17 (2.73-3.68)*	2.02 (1.72-2.37)*	1.56 (1.31-1.85)*	3.00 (2.61-3.46)*	1.99 (1.71-2.32)*	1.47 (1.25-1.73)*
Weakness and asymmetry	3.84 (3.06-4.82)*	2.35 (1.85-2.98)*	1.65 (1.28-2.14)*	3.77 (2.97-4.78)*	2.25 (1.78-2.89)*	1.54 (1.18-2.03)†	4.38 (3.49-5.45)*	2.73 (2.16-3.47)*	1.92 (1.48-2.49)*
**HGS asymmetry ratio at 30%**									
HGS asymmetry									
No	Ref	Ref	Ref	Ref	Ref	Ref	Ref	Ref	Ref
Yes	2.57 (2.14-3.08)*	2.05 (1.70-2.49)*	1.77 (1.44-2.18)*	2.98 (2.45-3.61)*	2.37 (1.94-2.91)*	2.03 (1.63-2.54)*	2.63 (2.19-3.18)*	2.08 (1.71-2.53)*	1.77 (1.43-2.19)*
Weakness and asymmetry combined groups									
Normal and symmetric HGS	Ref	Ref	Ref	Ref	Ref	Ref	Ref	Ref	Ref
Asymmetry only	2.11 (1.69-2.64)*	1.82 (1.45-2.30)*	1.64 (1.28-2.11)*	2.78 (2.18-3.55)*	2.43 (1.89-3.12)*	2.20 (1.68-2.89)*	2.12 (1.67-2.68)*	1.81 (1.41-2.30)*	1.59 (1.23-2.08)*
Weakness only	2.88 (2.54-3.27)*	1.87 (1.63-2.14)*	1.40 (1.21-1.62)*	3.06 (2.66-3.52)*	1.95 (1.68-2.26)*	1.49 (1.27-1.75)*	2.96 (2.59-3.37)*	1.96 (1.70-2.56)*	1.45 (1.24-1.69)*
Weakness and asymmetry	5.60 (4.04-7.77)*	3.46 (2.46-4.87)*	2.41 (1.67-3.46)*	5.19 (3.77-7.13)*	3.12 (2.23-4.35)*	2.16 (1.51-3.09)*	5.92 (4.31-8.13)*	3.68 (2.65-5.13)*	2.54 (1.79-3.63)*

HGS – handgrip strength, ADL – activities of daily living, IADL – instrumental activities of daily living, OR – odds ratio, CI – confidence interval.

Model 1: crude model; Model 2: adjusted for age and sex; Model 3: adjusted as model 2 plus marital status, educational level, residence, household income, occupation, social activities, smoking, drinking, multimorbidity, visual and hearing problems, depression score, cognitive function, and BMI.

*P<0.001.

†P<0.01.

Table S7. Cross-sectional association between HGS status and disability scores

Groups	Functional disability score			ADL disability score			IADL disability score		
	**Model 1 β (95% CI)**	**Model 2 β (95% CI)**	**Model 3 β (95% CI)**	**Model 1 β (95% CI)**	**Model 2 β (95% CI)**	**Model 3 β (95% CI)**	**Model 1 β (95% CI)**	**Model 2 β (95% CI)**	**Model 3 β (95% CI)**
HGS weakness									
No	Ref	Ref	Ref	Ref	Ref	Ref	Ref	Ref	Ref
Yes	2.42 (2.25-2.59)*	1.79 (1.62-1.97)*	1.31 (1.14-1.48)*	0.89 (0.82-0.96)*	0.69 (0.61-0.76)*	0.53 (0.45-0.60)*	1.53 (1.41-1.65)*	1.11 (0.99-1.23)*	0.78 (0.66-0.89)*
HGS asymmetry									
No	Ref	Ref	Ref	Ref	Ref	Ref	Ref	Ref	Ref
Yes	0.45 (0.34-0.56)*	0.31 (0.21-0.42)*	0.19 (0.09-0.29)*	0.15 (0.10-0.20)*	0.11 (0.06-0.15)*	0.07 (0.02-0.11)†	0.29 (0.22-0.37)*	0.20 (0.13-0.28)*	0.13 (0.06-0.19)*
Weakness and asymmetry combined groups									
Normal and symmetric HGS	Ref	Ref	Ref	Ref	Ref	Ref	Ref	Ref	Ref
Asymmetry only	0.25 (0.13-0.36)*	0.16 (0.05-0.27)†	0.08 (-0.03, 0.18)	0.08 (0.03-0.13)†	0.05 (0.004-0.10)‡	0.02 (-0.03, 0.07)	0.17 (0.09-0.24)*	0.11 (0.03-0.19)†	0.05 (-0.02, 0.12)
Weakness only	2.02 (1.77-2.27)*	1.38 (1.13-1.63)*	0.93 (0.70-1.17)*	0.75 (0.64-0.85)*	0.54 (0.43-0.65)*	0.39 (0.29-0.50)*	1.28 (1.11-1.45)*	0.85 (0.67-1.02)*	0.54 (0.38-0.71)*
Weakness and asymmetry	2.94 (2.70-3.17)*	2.27 (2.04-2.51)*	1.68 (1.46-1.91)*	1.07 (0.97-1.16)*	0.85 (0.75-0.95)*	0.66 (0.56-0.76)*	1.87 (1.71-2.02)*	1.41 (1.25-1.58)*	1.02 (0.86-1.17)*

HGS – handgrip strength, ADL – activities of daily living, IADL – instrumental activities of daily living, CI – confidence interval.

Model 1: crude model; Model 2: adjusted for age and sex; Model 3: adjusted as model 2 plus marital status, educational level, residence, household income, occupation, social activities, smoking, drinking, multimorbidity, visual and hearing problems, depression score, cognitive function, and BMI.

**P*<0.001.

†*P*<0.01.

‡*P*<0.05.

Table S8. Cross-sectional association between HGS status and functional dependency in subpopulations of 5288 participants with complete data

Groups	Low dependency (vs Independent)	Medium dependency (vs Independent)	High dependency (vs Independent)
	OR (95% CI)	OR (95% CI)	OR (95% CI)
HGS weakness			
No	Ref	Ref	Ref
Yes	1.35 (0.95-1.92)	1.24 (0.84-1.83)	1.41 (1.09-1.82)†
HGS asymmetry			
No	Ref	Ref	Ref
Yes	0.97 (0.77-1.22)	1.10 (0.85-1.42)	1.33 (1.12-1.59)*
Weakness and asymmetry combined groups			
Normal and symmetric HGS	Ref	Ref	Ref
Asymmetry only	0.93 (0.72-1.20)	1.09 (0.82-1.44)	1.28 (1.06-1.54)†
Weakness only	1.23 (0.75-2.01)	1.21 (0.69-2.12)	1.22 (0.83-1.79)
Weakness and asymmetry	1.39 (0.87-2.22)	1.36 (0.82-2.27)	1.91 (1.37-2.67)*

HGS – handgrip strength, OR – odds ratio, CI – confidence interval.

Adjusted for age, sex, marital status, educational level, residence, household income, occupation, social activities, smoking, drinking, multimorbidity, visual and hearing problems, depression score, cognitive function, and BMI.

*P<0.001.

†P<0.01.

Table S9. Cross-sectional association between HGS status and functional dependency, with HGS asymmetry defined by 20% and 30% rule

Groups	Low dependency (vs Independent)	Medium dependency (vs Independent)	High dependency (vs Independent)
	**OR (95% CI)**	**OR (95% CI)**	**OR (95% CI)**
**HGS asymmetry ratio at 20%**			
HGS asymmetry			
No	Ref	Ref	Ref
Yes	1.26 (1.02-1.56)‡	1.24 (0.99-1.56)	1.29 (1.01-1.51)†
Weakness and asymmetry combined groups			
Normal and symmetric HGS	Ref	Ref	Ref
Asymmetry only	1.17 (0.91-1.49)	1.23 (0.94-1.60)	1.32 (1.10-1.59)†
Weakness only	1.20 (0.92-1.56)	1.32 (1.01-1.73)‡	1.65 (1.38-1.99)*
Weakness and asymmetry	1.75 (1.18-2.59)†	1.49 (0.99-2.27)	1.65 (1.22-2.23)†
**HGS asymmetry ratio at 30%**			
HGS asymmetry			
No	Ref	Ref	Ref
Yes	1.37 (0.97-1.94)	1.32 (0.91-1.93)	2.15 (1.70-2.73)*
Weakness and asymmetry combined groups			
Normal and symmetric HGS	Ref	Ref	Ref
Asymmetry only	0.95 (0.58-1.54)	1.03 (0.61-1.72)	2.32 (1.74-3.08)*
Weakness only	1.18 (0.92-1.51)	1.25 (0.97-1.61)	1.57 (1.32-1.87)*
Weakness and asymmetry	2.46 (1.47-4.14)*	2.07 (1.19-3.62)†	2.50 (1.65-3.77)*

HGS – handgrip strength, OR – odds ratio, CI – confidence interval.

Adjusted for age, sex, marital status, educational level, residence, household income, occupation, social activities, smoking, drinking, multimorbidity, visual and hearing problems, depression score, cognitive function, and BMI.

**P*<0.001.

†*P*<0.01.

‡*P*<0.05.

Table S10. Longitudinal association of HGS status with functional disability in subpopulations of 3452 participants with complete data

Groups	Functional disability			ADL disability			IADL disability		
	**Model 1 OR (95% CI)**	**Model 2 OR (95% CI)**	**Model 3 OR (95% CI)**	**Model 1 OR (95% CI)**	**Model 2 OR (95% CI)**	**Model 3 OR (95% CI)**	**Model 1 OR (95% CI)**	**Model 2 OR (95% CI)**	**Model 3 OR (95% CI)**
HGS weakness									
No	Ref	Ref	Ref	Ref	Ref	Ref	Ref	Ref	Ref
Yes	2.66 (2.20-3.20)*	1.96 (1.61-2.38)*	1.89 (1.41-2.53)*	2.49 (2.03-3.06)*	1.82 (1.47-2.26)*	1.70 (1.24-2.33)*	2.56 (2.10-3.11)*	1.85 (1.51-2.28)*	1.70 (1.25-2.33)*
HGS asymmetry									
No	Ref	Ref	Ref	Ref	Ref	Ref	Ref	Ref	Ref
Yes	1.29 (1.16-1.43)*	1.20 (1.07-1.34)*	1.29 (1.09-1.52)†	1.29 (1.14-1.47)*	1.20 (1.05-1.37)†	1.25 (1.03-1.52)‡	1.31 (1.17-1.48)*	1.21 (1.07-1.37)†	1.22 (1.01-1.48)‡
Weakness and asymmetry combined groups									
Normal and symmetric HGS	Ref	Ref	Ref	Ref	Ref	Ref	Ref	Ref	Ref
Asymmetry only	1.24 (1.10-1.38)*	1.16 (1.04-1.31)†	1.24 (1.04-1.48)‡	1.19 (1.04-1.37)‡	1.13 (0.98-1.30)	1.20 (0.97-1.48)	1.27 (1.12-1.44)*	1.19 (1.04-1.36)†	1.18 (0.96-1.44)
Weakness only	2.49 (1.91-3.26)*	1.86 (1.41-2.45)*	1.67 (1.11-2.53)‡	1.97 (1.44-2.70)*	1.45 (1.05-2.00)‡	1.49 (0.94-2.36)	2.49 (1.87-3.32)*	1.83 (1.36-2.46)*	1.49 (0.95-2.34)
Weakness and asymmetry	3.31 (2.56-4.26)*	2.32 (1.78-3.02)*	2.53 (1.70-3.77)*	3.43 (2.62-4.50)*	2.41 (1.82-3.19)*	2.21 (1.44-3.38)*	3.14 (2.40-4.10)*	2.15 (1.63-2.84)*	2.19 (1.44-3.33)*

HGS – handgrip strength, ADL – activities of daily living, IADL – instrumental activities of daily living, OR – odds ratio, CI – confidence interval.

Model 1: crude model; Model 2: adjusted for age and sex; Model 3: adjusted as model 2 plus marital status, educational level, residence, household income, occupation, social activities, smoking, drinking, multimorbidity, visual and hearing problems, depression score, cognitive function, BMI, and follow-up time.

*$P<0.001$.

†$P<0.01$.

‡$P<0.05$.

Table S11. Longitudinal association between HGS status and functional disability, with HGS asymmetry defined by 20% and 30% rule

Groups	Functional disability			ADL disability			IADL disability		
	**Model 1 OR (95% CI)**	**Model 2 OR (95% CI)**	**Model 3 OR (95% CI)**	**Model 1 OR (95% CI)**	**Model 2 OR (95% CI)**	**Model 3 OR (95% CI)**	**Model 1 OR (95% CI)**	**Model 2 OR (95% CI)**	**Model 3 OR (95% CI)**
**HGS asymmetry ratio at 20%**									
HGS asymmetry									
No	Ref	Ref	Ref	Ref	Ref	Ref	Ref	Ref	Ref
Yes	1.44 (1.22-1.69)*	1.28 (1.09-1.51)†	1.22 (1.03-1.46)‡	1.54 (1.28-1.85)*	1.39 (1.15-1.68)*	1.33 (1.09-1.62)†	1.39 (1.16-1.67)*	1.23 (1.02-1.48)‡	1.16 (0.96-1.41)
Weakness and asymmetry combined groups									
Normal and symmetric HGS	Ref	Ref	Ref	Ref	Ref	Ref	Ref	Ref	Ref
Asymmetry only	1.29 (1.08-1.54)†	1.18 (0.98-1.42)	1.13 (0.93-1.37)	1.32 (1.07-1.63)‡	1.22 (0.98-1.51)	1.17 (0.94-1.47)	1.30 (1.06-1.58)‡	1.18 (0.96-1.45)	1.12 (0.90-1.38)
Weakness only	2.49 (2.02-3.07)*	1.84 (1.48-2.29)*	1.47 (1.17-1.85)*	2.18 (1.72-2.77)*	1.59 (1.24-2.04)*	1.28 (0.99-1.66)	2.53 (2.03-3.17)*	1.84 (1.46-2.33)*	1.46 (1.15-1.87)†
Weakness and asymmetry	3.69 (2.55-5.36)*	2.61 (1.78-3.84)*	2.19 (1.47-3.27)*	4.17 (2.84-6.11)*	2.98 (2.01-4.42)*	2.50 (1.67-3.76)*	2.97 (2.01-4.38)*	2.04 (1.37-3.05)*	1.70 (1.12-2.57)‡
**HGS asymmetry ratio at 30%**									
HGS asymmetry									
No	Ref	Ref	Ref	Ref	Ref	Ref	Ref	Ref	Ref
Yes	1.82 (1.38-2.41)*	1.53 (1.15-2.04)†	1.41 (1.04-1.91)‡	1.89 (1.36-2.55)*	1.59 (1.15-2.20)†	1.45 (1.04-2.03)‡	1.74 (1.28-2.37)*	1.45 (1.06-1.99)‡	1.33 (0.96-1.85)
Weakness and asymmetry combined groups									
Normal and symmetric HGS	Ref	Ref	Ref	Ref	Ref	Ref	Ref	Ref	Ref
Asymmetry only	1.77 (1.28-2.44)*	1.53 (1.10-2.13)‡	1.46 (1.03-2.06)‡	1.56 (1.07-2.29)‡	1.37 (0.93-2.02)	1.29 (0.86-1.93)	1.77 (1.25-2.52)*	1.53 (1.07-2.20)‡	1.46 (1.00-2.12)
Weakness only	2.69 (2.22-3.27)*	1.99 (1.62-2.44)*	1.62 (1.31-2.01)*	2.39 (1.92-2.97)*	1.74 (1.37-2.18)*	1.43 (1.13-1.81)†	2.63 (2.14-3.24)*	1.91 (1.54-2.37)*	1.55 (1.24-1.94)*
Weakness and asymmetry	2.78 (1.58-4.89)*	1.96 (1.09-3.51)‡	1.52 (0.83-2.79)	3.98 (2.23-7.10)*	2.91 (1.61-5.28)*	2.28 (1.23-4.23)†	2.31 (1.25-4.25)†	1.59 (0.85-2.98)	1.23 (0.64-2.34)

HGS – handgrip strength, ADL – activities of daily living, IADL – instrumental activities of daily living, OR – odds ratio, CI – confidence interval.

Model 1: crude model; Model 2: adjusted for age and sex; Model 3: adjusted as model 2 plus marital status, educational level, residence, household income, occupation, social activities, smoking, drinking, multimorbidity, visual and hearing problems, depression score, cognitive function, BMI, and follow-up time.

*P<0.001.

†P<0.01.

‡P<0.05.

Table S12. Longitudinal association of HGS status with disability scores

Groups	Functional disability score			ADL disability score			IADL disability score		
	**Model 1 β (95% CI)**	**Model 2 β (95% CI)**	**Model 3 β (95% CI)**	**Model 1 β (95% CI)**	**Model 2 β (95% CI)**	**Model 3 β (95% CI)**	**Model 1 β (95% CI)**	**Model 2 β (95% CI)**	**Model 3 β (95% CI)**
HGS weakness									
No	Ref	Ref	Ref	Ref	Ref	Ref	Ref	Ref	Ref
Yes	1.69 (1.42-1.95)*	1.23 (0.96-1.50)*	1.02 (0.75-1.28)*	0.65 (0.53-0.77)*	0.48 (0.36-0.60)*	0.41 (0.29-0.53)*	1.04 (0.86-1.21)*	0.74 (0.57-0.92)*	0.60 (0.43-0.77)*
HGS asymmetry									
No	Ref	Ref	Ref	Ref	Ref	Ref	Ref	Ref	Ref
Yes	0.34 (0.20-0.47)*	0.25 (0.12-0.39)*	0.22 (0.08-0.35)*	0.11 (0.05-0.17)*	0.08 (0.02-0.14)‡	0.06 (0.003, 0.12)‡	0.24 (0.15-0.33)*	0.18 (0.10-0.27)*	0.16 (0.07-0.24)*
Weakness and asymmetry combined groups									
Normal and symmetric HGS	Ref	Ref	Ref	Ref	Ref	Ref	Ref	Ref	Ref
Asymmetry only	0.22 (0.08-0.36)†	0.16 (0.02-0.29)‡	0.13 (-0.01, 0.26)	0.05 (-0.01, 0.12)	0.03 (-0.03, 0.09)	0.02 (-0.04, 0.08)	0.17 (0.08-0.26)*	0.13 (0.04-0.22)†	0.12 (0.02-0.20)‡
Weakness only	1.19 (0.81-1.57)*	0.75 (0.37-1.12)*	0.54 (0.16-0.91)†	0.41 (0.24-0.58)*	0.25 (0.08-0.42)†	0.18 (0.01-0.35)‡	0.76 (0.51-1.00)*	0.48 (0.23-0.72)*	0.33 (0.09-0.57)†
Weakness and asymmetry	2.30 (1.94-2.67)*	1.80 (1.44-2.17)*	1.56 (1.20-1.93)*	0.90 (0.74-1.07)*	0.72 (0.56-0.89)*	0.64 (0.47-0.80)*	1.42 (1.18-1.65)*	1.10 (0.86-1.33)*	0.93 (0.70-1.17)*

HGS – handgrip strength, ADL – activities of daily living, IADL – instrumental activities of daily living, CI – confidence interval.

Model 1: crude model; Model 2: adjusted for age and sex; Model 3: adjusted as model 2 plus marital status, educational level, residence, household income, occupation, social activities, smoking, drinking, multimorbidity, visual and hearing problems, depression score, cognitive function, BMI, and follow-up time.

**P*<0.001.

†*P*<0.01.

‡*P*<0.05.

Table S13. Longitudinal association between HGS status and functional dependency in subpopulations of 3452 participants with complete data

Groups	Low dependency (vs Independent)	Medium dependency (vs Independent)	High dependency (vs Independent)
	**OR (95% CI)**	**OR (95% CI)**	**OR (95% CI)**
HGS weakness			
No	Ref	Ref	Ref
Yes	1.48 (0.87-2.50)	2.36 (1.36-4.09)†	1.90 (1.35-2.66)*
HGS asymmetry			
No	Ref	Ref	Ref
Yes	1.21 (0.91-1.62)	1.52 (1.05-2.21)‡	1.28 (1.04-1.57)‡
Weakness and asymmetry combined groups			
Normal and symmetric HGS	Ref	Ref	Ref
Asymmetry only	1.10 (0.81-1.49)	1.64 (1.09-2.45)‡	1.24 (1.00-1.54)
Weakness only	0.75 (0.29-1.93)	3.04 (1.46-6.32)†	1.75 (1.09-2.82)†
Weakness and asymmetry	2.46 (1.30-4.67)†	2.84 (1.28-6.31)‡	2.44 (1.54-3.88)*

HGS – handgrip strength, OR – odds ratio, CI – confidence interval.

Adjusted for age, sex, marital status, educational level, residence, household income, occupation, social activities, smoking, drinking, multimorbidity, visual and hearing problems, depression score, cognitive function, BMI, and follow-up time.

**P*<0.001.

†*P*<0.01.

‡*P*<0.05.

Table S14. Longitudinal association between HGS status and functional dependency, with HGS asymmetry defined by 20% and 30% rule

**Groups**	**Low dependency (vs Independent)**	**Medium dependency (vs Independent)**	**High dependency (vs Independent)**
	**OR (95% CI)**	**OR (95% CI)**	**OR (95% CI)**
**HGS asymmetry ratio at 20%**			
HGS asymmetry			
No	Ref	Ref	Ref
Yes	0.97 (0.71-1.31)	1.39 (0.98-1.99)	1.31 (1.07-1.62)†
Weakness and asymmetry combined groups			
Normal and symmetric HGS	Ref	Ref	Ref
Asymmetry only	0.92 (0.66-1.29)	1.42 (0.97-2.10)	1.18 (0.93-1.49)
Weakness only	1.31 (0.89-1.93)	1.71 (1.10-2.66)‡	1.49 (1.14-1.95)†
Weakness and asymmetry	1.45 (0.72-2.92)	1.83 (0.80-4.15)	2.54 (1.63-3.96)*
**HGS asymmetry ratio at 30%**			
HGS asymmetry			
No	Ref	Ref	Ref
Yes	1.31 (0.80-2.14)	1.05 (0.52-2.12)	1.57 (1.10-2.23)‡
Weakness and asymmetry combined groups			
Normal and symmetric HGS	Ref	Ref	Ref
Asymmetry only	1.42 (0.82-2.45)	1.55 (0.77-3.15)	1.48 (0.97-2.25)
Weakness only	1.40 (0.98-2.01)	1.88 (1.26-2.80)†	1.64 (1.28-2.10)*
Weakness and asymmetry	1.11 (0.38-3.26)	NA	2.09 (1.09-3.99)‡

HGS – handgrip strength, OR – odds ratio, CI – confidence interval, NA – not available due to limited sample sizes.

Adjusted for age, sex, marital status, educational level, residence, household income, occupation, social activities, smoking, drinking, multimorbidity, visual and hearing problems, depression score, cognitive function, BMI, and follow-up time.

* $P<0.001$.

† $P<0.01$.

‡ $P<0.05$.

Figure S1. Subgroup analyses for the longitudinal associations of HGS status with functional disability. HGS – handgrip strength, ADL – activities of daily living, IADL – instrumental activities of daily living, OR – odds ratio, CI – confidence interval, G1 – Normal and symmetric HGS, G2 – Asymmetry only, G3 – Weakness only, G4 – Weakness and asymmetry. Adjusted for age, sex, marital status, educational level, residence, household income, occupation, social activities, smoking, drinking, multimorbidity, visual and hearing problems, depression score, cognitive function, BMI, and follow-up time, except for the stratification factor. **P*<0.001. †*P*<0.01. ‡*P*<0.05.

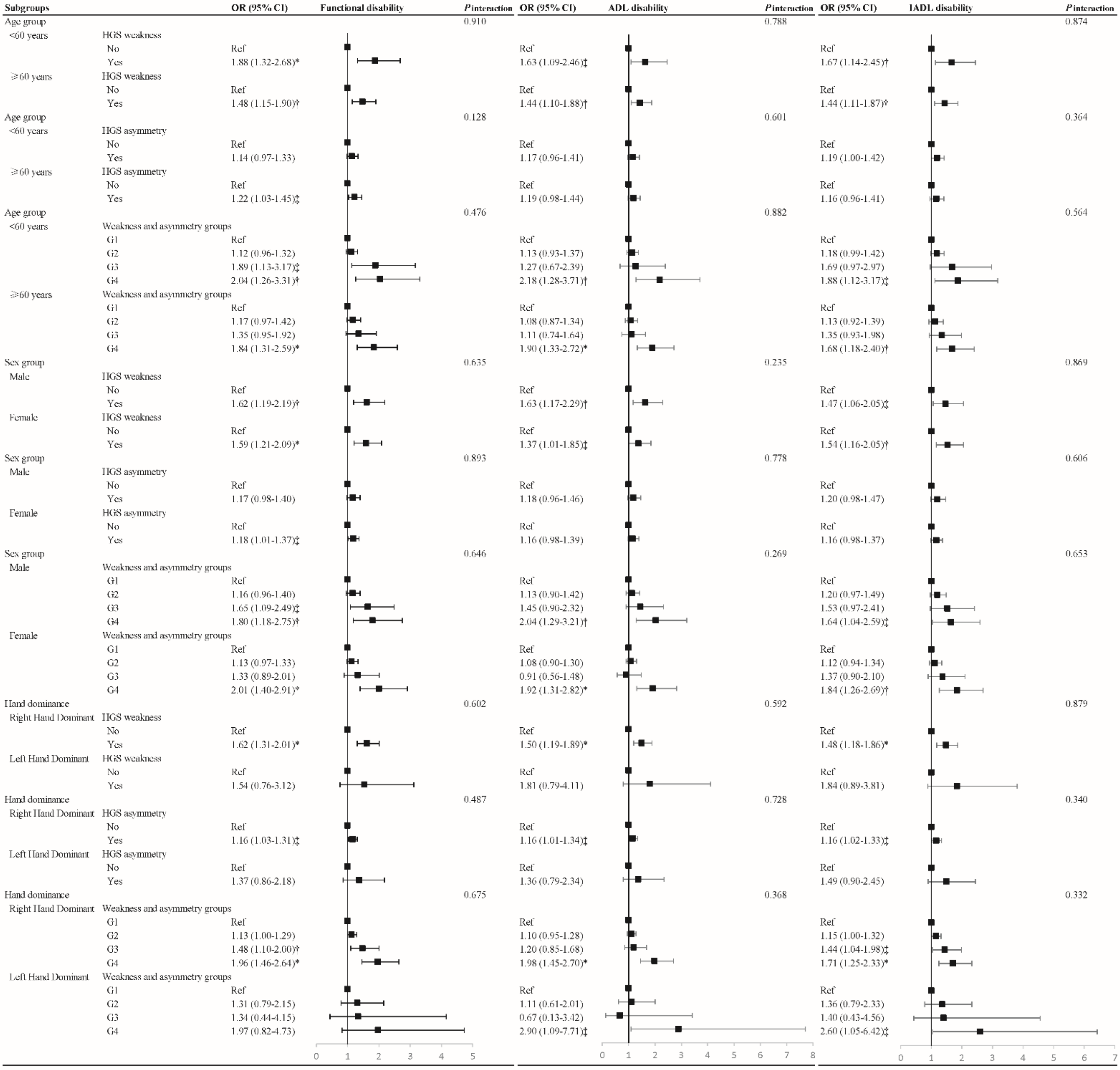

Figure S2. Subgroup analyses for the longitudinal association of HGS status with functional dependency. HGS – handgrip strength, OR – odds ratio, CI – confidence interval, G1 – Normal and symmetric HGS, G2 – Asymmetry only, G3 – Weakness only, G4 – Weakness and asymmetry. Adjusted for age, sex, marital status, educational level, residence, household income, occupation, social activities, smoking, drinking, multimorbidity, visual and hearing problems, depression score, cognitive function, BMI, and follow-up time, except for the stratification factor. *P<0.001. †P<0.01. ‡P<0.05.

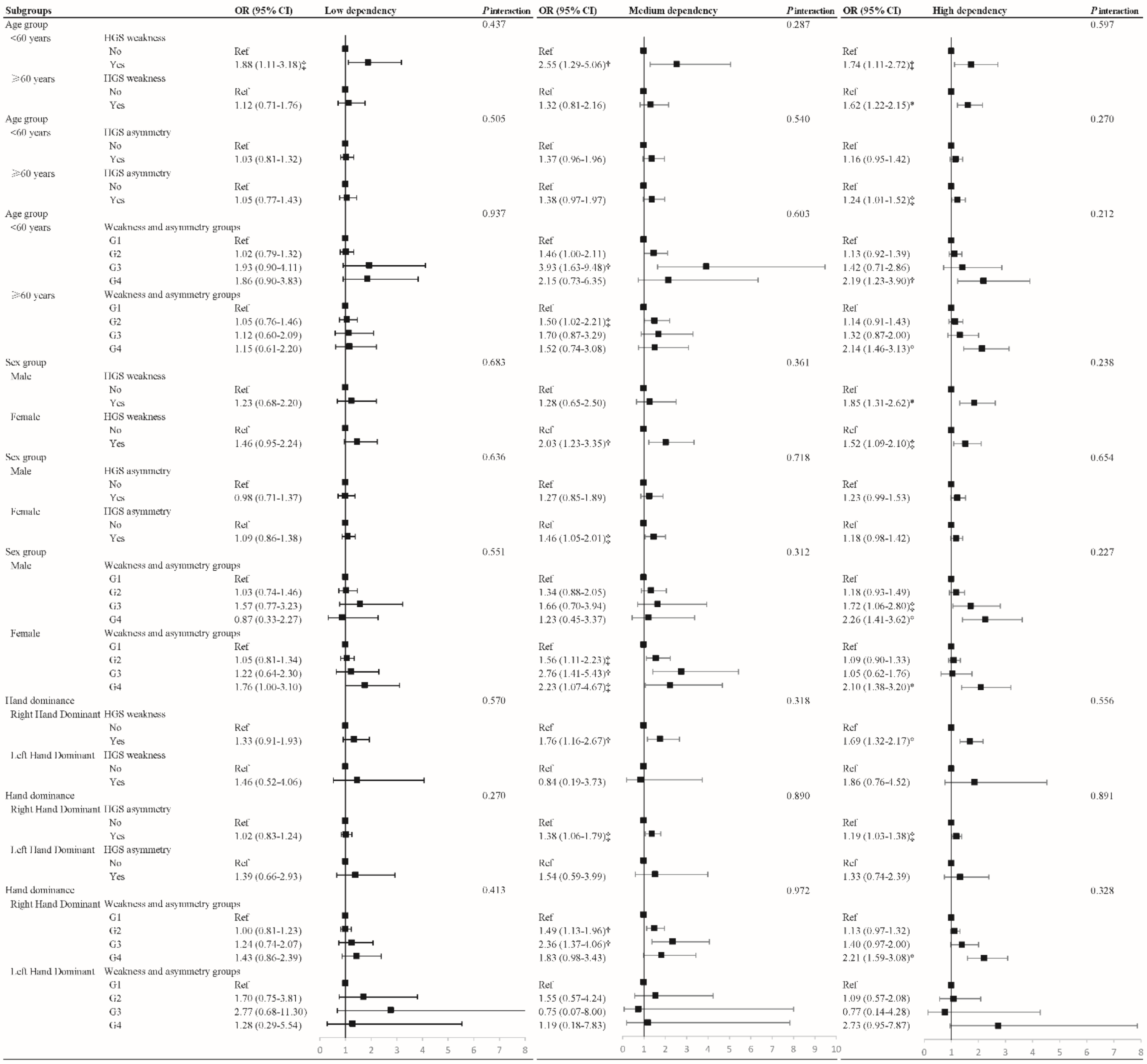

Supplement: Online Supplementary Document [file jogh-14-04047-s001.pdf]
